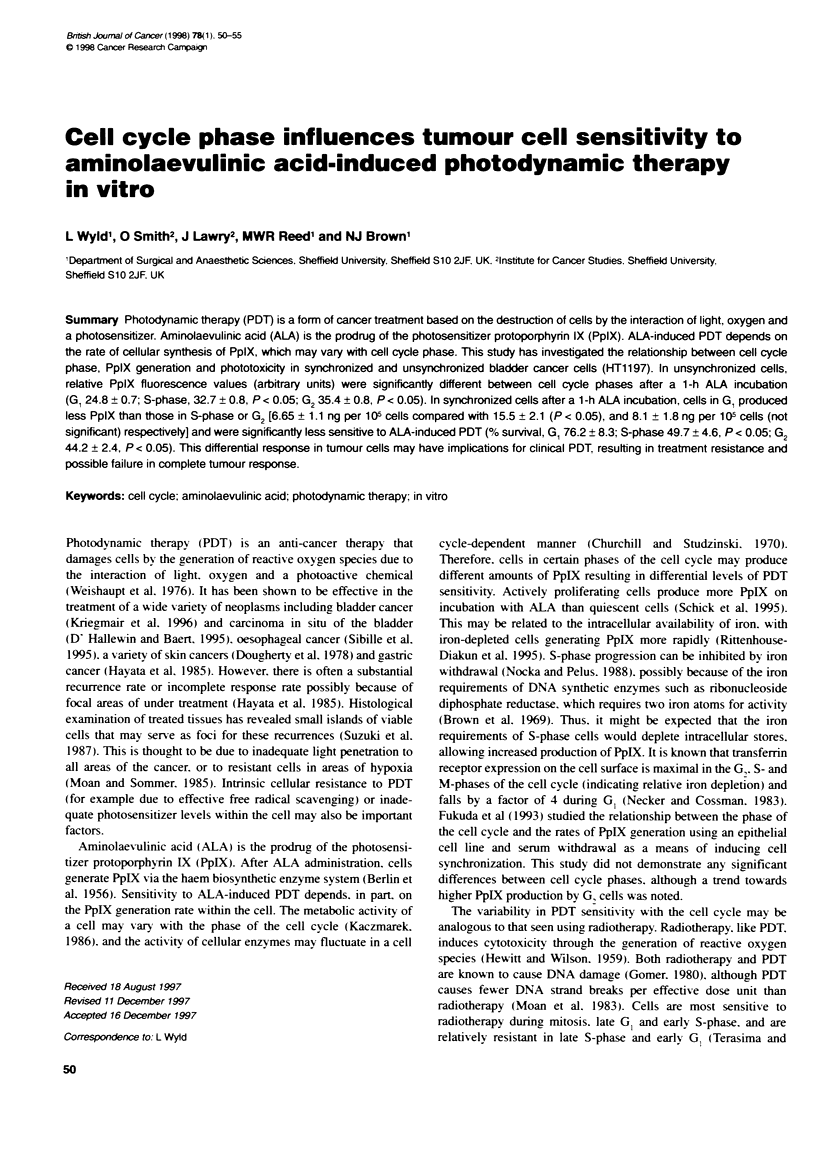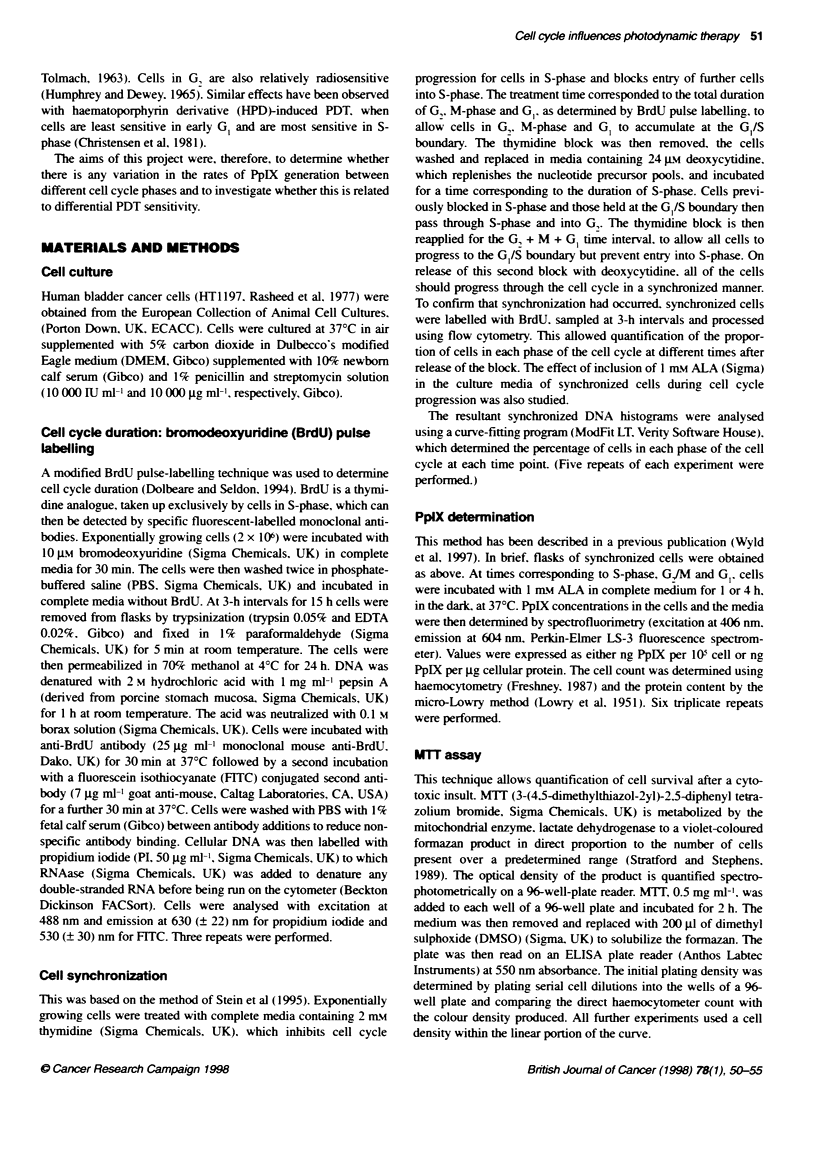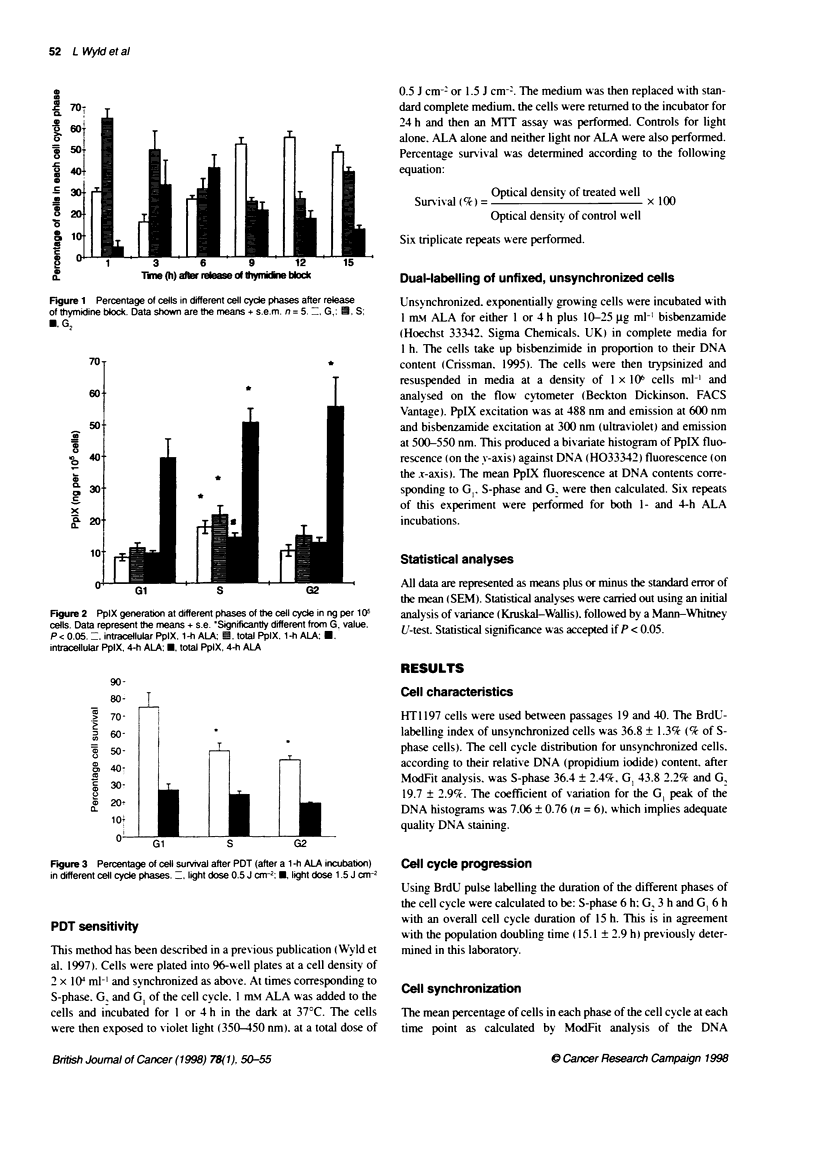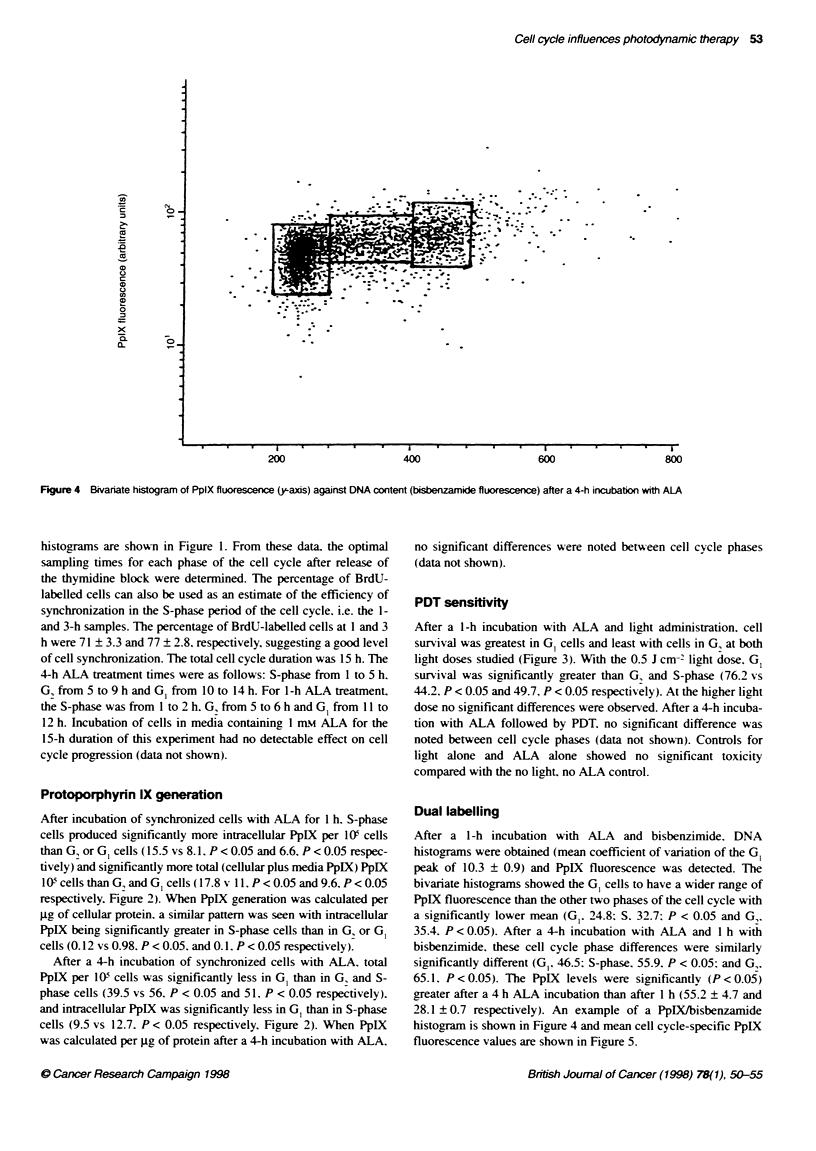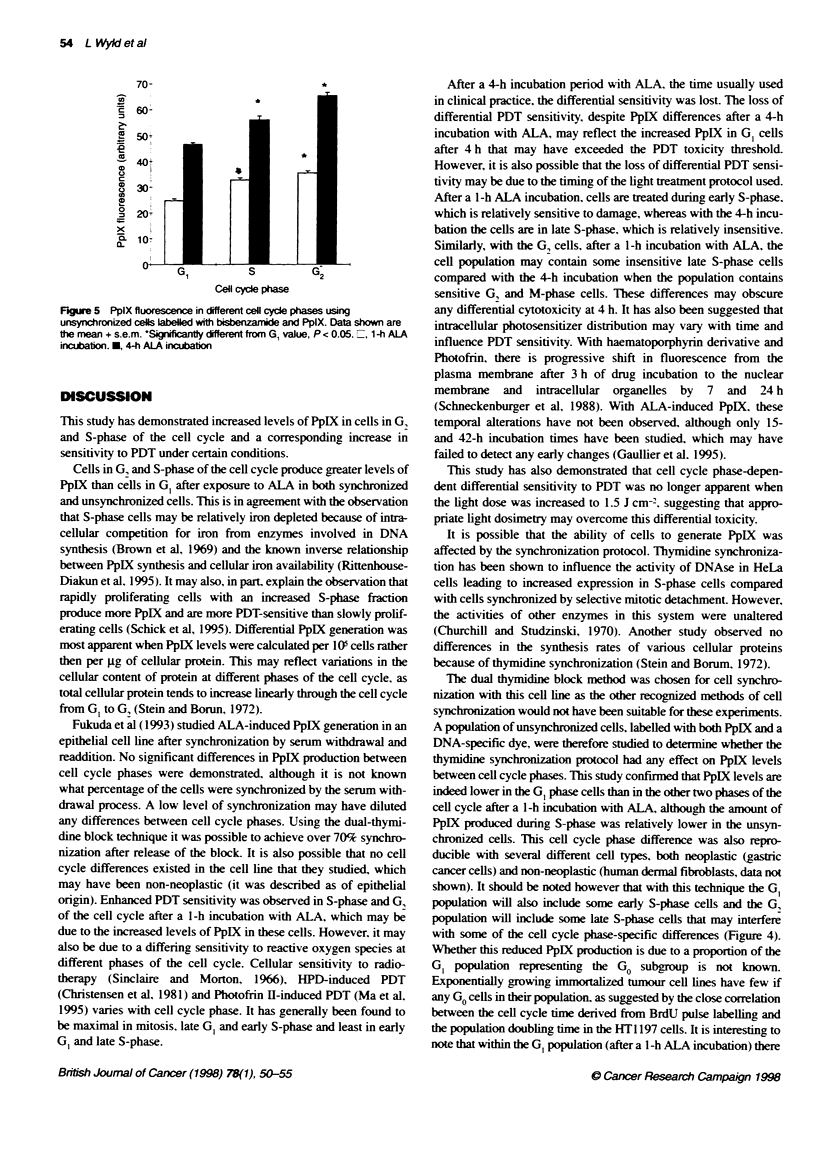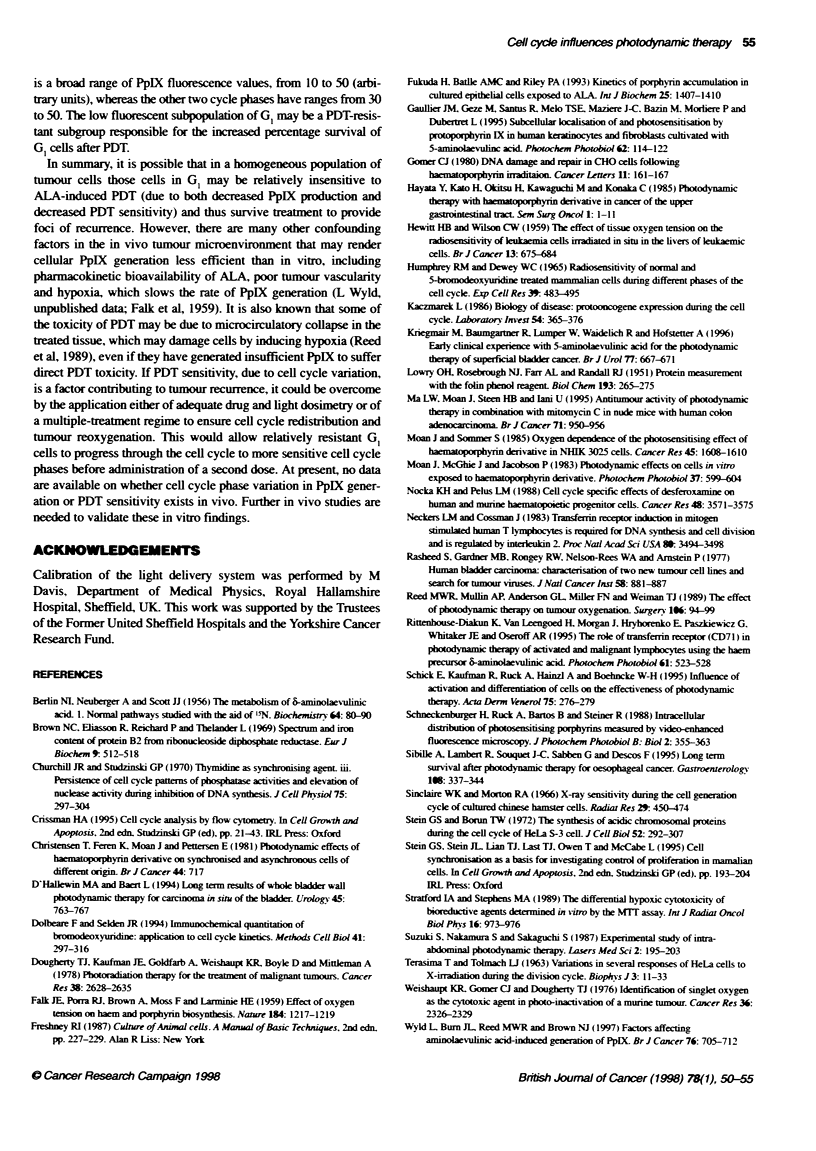# Cell cycle phase influences tumour cell sensitivity to aminolaevulinic acid-induced photodynamic therapy in vitro.

**DOI:** 10.1038/bjc.1998.441

**Published:** 1998-07

**Authors:** L. Wyld, O. Smith, J. Lawry, M. W. Reed, N. J. Brown

**Affiliations:** Department of Surgical and Anaesthetic Sciences, Sheffield University, UK.

## Abstract

Photodynamic therapy (PDT) is a form of cancer treatment based on the destruction of cells by the interaction of light, oxygen and a photosensitizer. Aminolaevulinic acid (ALA) is the prodrug of the photosensitizer protoporphyrin IX (PpIX). ALA-induced PDT depends on the rate of cellular synthesis of PpIX, which may vary with cell cycle phase. This study has investigated the relationship between cell cycle phase, PpIX generation and phototoxicity in synchronized and unsynchronized bladder cancer cells (HT1197). In unsynchronized cells, relative PpIX fluorescence values (arbitrary units) were significantly different between cell cycle phases after a 1-h ALA incubation (G1 24.8 +/- 0.7; S-phase, 32.7 +/- 0.8, P < 0.05; G2 35.4 +/- 0.8, P < 0.05). In synchronized cells after a 1-h ALA incubation, cells in G1 produced less PpIX than those in S-phase or G2 [6.65 +/- 1.1 ng per 10(5) cells compared with 15.5 +/- 2.1 (P < 0.05), and 8.1 +/- 1.8 ng per 10(5) cells (not significant) respectively] and were significantly less sensitive to ALA-induced PDT (% survival, G1 76.2 +/- 8.3; S-phase 49.7 +/- 4.6, P < 0.05; G2 44.2 +/- 2.4, P < 0.05). This differential response in tumour cells may have implications for clinical PDT, resulting in treatment resistance and possible failure in complete tumour response.


British Journal of Cancer (1998) 78(1). 50–55
© 1998 Cancer Research Campaign

### Cell cycle phase influences tumour cell sensitivity to aminolaevulinic acid-induced photodynamic therapy in vitro

L Wyld[1], O Smith[2], J Lawry[2], MWR Reed[1] and NJ Brown[1]

[1]Department of Surgical and Anaesthetic Sciences. Sheffield University. Sheffield S10 2JF. UK. [2]Institute for Cancer Studies. Sheffield University. Sheffield S10 2JF. UK

**Summary** Photodynamic therapy (PDT) is a form of cancer treatment based on the destruction of cells by the interaction of light, oxygen and a photosensitizer. Aminolaevulinic acid (ALA) is the prodrug of the photosensitizer protoporphyrin IX (PpIX). ALA-induced PDT depends on the rate of cellular synthesis of PpIX, which may vary with cell cycle phase. This study has investigated the relationship between cell cycle phase, PpIX generation and phototoxicity in synchronized and unsynchronized bladder cancer cells (HT1197). In unsynchronized cells, relative PpIX fluorescence values (arbitrary units) were significantly different between cell cycle phases after a 1-h ALA incubation ($G_1$ 24.8 ± 0.7; S-phase, 32.7 ± 0.8, $P < 0.05$; $G_2$ 35.4 ± 0.8, $P < 0.05$). In synchronized cells after a 1-h ALA incubation, cells in $G_1$ produced less PpIX than those in S-phase or $G_2$ [6.65 ± 1.1 ng per $10^5$ cells compared with 15.5 ± 2.1 ($P < 0.05$), and 8.1 ± 1.8 ng per $10^5$ cells (not significant) respectively] and were significantly less sensitive to ALA-induced PDT (% survival, $G_1$ 76.2 ± 8.3; S-phase 49.7 ± 4.6, $P < 0.05$; $G_2$ 44.2 ± 2.4, $P < 0.05$). This differential response in tumour cells may have implications for clinical PDT, resulting in treatment resistance and possible failure in complete tumour response.

**Keywords:** cell cycle; aminolaevulinic acid; photodynamic therapy; in vitro

Photodynamic therapy (PDT) is an anti-cancer therapy that damages cells by the generation of reactive oxygen species due to the interaction of light, oxygen and a photoactive chemical (Weishaupt et al, 1976). It has been shown to be effective in the treatment of a wide variety of neoplasms including bladder cancer (Kriegmair et al, 1996) and carcinoma in situ of the bladder (D' Hallewin and Baert, 1995), oesophageal cancer (Sibille et al, 1995), a variety of skin cancers (Dougherty et al, 1978) and gastric cancer (Hayata et al, 1985). However, there is often a substantial recurrence rate or incomplete response rate possibly because of focal areas of under treatment (Hayata et al, 1985). Histological examination of treated tissues has revealed small islands of viable cells that may serve as foci for these recurrences (Suzuki et al, 1987). This is thought to be due to inadequate light penetration to all areas of the cancer, or to resistant cells in areas of hypoxia (Moan and Sommer, 1985). Intrinsic cellular resistance to PDT (for example due to effective free radical scavenging) or inadequate photosensitizer levels within the cell may also be important factors.

Aminolaevulinic acid (ALA) is the prodrug of the photosensitizer protoporphyrin IX (PpIX). After ALA administration, cells generate PpIX via the haem biosynthetic enzyme system (Berlin et al, 1956). Sensitivity to ALA-induced PDT depends, in part, on the PpIX generation rate within the cell. The metabolic activity of a cell may vary with the phase of the cell cycle (Kaczmarek, 1986), and the activity of cellular enzymes may fluctuate in a cell cycle-dependent manner (Churchill and Studzinski, 1970). Therefore, cells in certain phases of the cell cycle may produce different amounts of PpIX resulting in differential levels of PDT sensitivity. Actively proliferating cells produce more PpIX on incubation with ALA than quiescent cells (Schick et al, 1995). This may be related to the intracellular availability of iron, with iron-depleted cells generating PpIX more rapidly (Rittenhouse-Diakun et al, 1995). S-phase progression can be inhibited by iron withdrawal (Nocka and Pelus, 1988), possibly because of the iron requirements of DNA synthetic enzymes such as ribonucleoside diphosphate reductase, which requires two iron atoms for activity (Brown et al, 1969). Thus, it might be expected that the iron requirements of S-phase cells would deplete intracellular stores, allowing increased production of PpIX. It is known that transferrin receptor expression on the cell surface is maximal in the $G_2$, S- and M-phases of the cell cycle (indicating relative iron depletion) and falls by a factor of 4 during $G_1$ (Necker and Cossman, 1983). Fukuda et al (1993) studied the relationship between the phase of the cell cycle and the rates of PpIX generation using an epithelial cell line and serum withdrawal as a means of inducing cell synchronization. This study did not demonstrate any significant differences between cell cycle phases, although a trend towards higher PpIX production by $G_2$ cells was noted.

The variability in PDT sensitivity with the cell cycle may be analogous to that seen using radiotherapy. Radiotherapy, like PDT, induces cytotoxicity through the generation of reactive oxygen species (Hewitt and Wilson, 1959). Both radiotherapy and PDT are known to cause DNA damage (Gomer, 1980), although PDT causes fewer DNA strand breaks per effective dose unit than radiotherapy (Moan et al, 1983). Cells are most sensitive to radiotherapy during mitosis, late $G_1$ and early S-phase, and are relatively resistant in late S-phase and early $G_1$ (Terasima and

*Received 18 August 1997*
*Revised 11 December 1997*
*Accepted 16 December 1997*

*Correspondence to:* L Wyld

Tolmach, 1963). Cells in $G_2$ are also relatively radiosensitive (Humphrey and Dewey, 1965). Similar effects have been observed with haematoporphyrin derivative (HPD)-induced PDT, when cells are least sensitive in early $G_1$ and are most sensitive in S-phase (Christensen et al, 1981).

The aims of this project were, therefore, to determine whether there is any variation in the rates of PpIX generation between different cell cycle phases and to investigate whether this is related to differential PDT sensitivity.

### MATERIALS AND METHODS

### Cell culture

Human bladder cancer cells (HT1197, Rasheed et al, 1977) were obtained from the European Collection of Animal Cell Cultures, (Porton Down, UK, ECACC). Cells were cultured at 37°C in air supplemented with 5% carbon dioxide in Dulbecco's modified Eagle medium (DMEM, Gibco) supplemented with 10% newborn calf serum (Gibco) and 1% penicillin and streptomycin solution (10 000 IU $ml^{-1}$ and 10 000 μg $ml^{-1}$, respectively, Gibco).

### Cell cycle duration: bromodeoxyuridine (BrdU) pulse labelling

A modified BrdU pulse-labelling technique was used to determine cell cycle duration (Dolbeare and Seldon, 1994). BrdU is a thymidine analogue, taken up exclusively by cells in S-phase, which can then be detected by specific fluorescent-labelled monoclonal antibodies. Exponentially growing cells ($2 \times 10^6$) were incubated with 10 μM bromodeoxyuridine (Sigma Chemicals, UK) in complete media for 30 min. The cells were then washed twice in phosphate-buffered saline (PBS, Sigma Chemicals, UK) and incubated in complete media without BrdU. At 3-h intervals for 15 h cells were removed from flasks by trypsinization (trypsin 0.05% and EDTA 0.02%, Gibco) and fixed in 1% paraformaldehyde (Sigma Chemicals, UK) for 5 min at room temperature. The cells were then permeabilized in 70% methanol at 4°C for 24 h. DNA was denatured with 2 M hydrochloric acid with 1 mg $ml^{-1}$ pepsin A (derived from porcine stomach mucosa, Sigma Chemicals, UK) for 1 h at room temperature. The acid was neutralized with 0.1 M borax solution (Sigma Chemicals, UK). Cells were incubated with anti-BrdU antibody (25 μg $ml^{-1}$ monoclonal mouse anti-BrdU, Dako, UK) for 30 min at 37°C followed by a second incubation with a fluorescein isothiocyanate (FITC) conjugated second antibody (7 μg $ml^{-1}$ goat anti-mouse, Caltag Laboratories, CA, USA) for a further 30 min at 37°C. Cells were washed with PBS with 1% fetal calf serum (Gibco) between antibody additions to reduce non-specific antibody binding. Cellular DNA was then labelled with propidium iodide (PI, 50 μg $ml^{-1}$, Sigma Chemicals, UK) to which RNAase (Sigma Chemicals, UK) was added to denature any double-stranded RNA before being run on the cytometer (Beckton Dickinson FACSort). Cells were analysed with excitation at 488 nm and emission at 630 (± 22) nm for propidium iodide and 530 (± 30) nm for FITC. Three repeats were performed.

### Cell synchronization

This was based on the method of Stein et al (1995). Exponentially growing cells were treated with complete media containing 2 mM thymidine (Sigma Chemicals, UK), which inhibits cell cycle progression for cells in S-phase and blocks entry of further cells into S-phase. The treatment time corresponded to the total duration of $G_2$, M-phase and $G_1$, as determined by BrdU pulse labelling, to allow cells in $G_2$, M-phase and $G_1$ to accumulate at the $G_1$/S boundary. The thymidine block was then removed, the cells washed and replaced in media containing 24 μM deoxycytidine, which replenishes the nucleotide precursor pools, and incubated for a time corresponding to the duration of S-phase. Cells previously blocked in S-phase and those held at the $G_1$/S boundary then pass through S-phase and into $G_2$. The thymidine block is then reapplied for the $G_2$ + M + $G_1$ time interval, to allow all cells to progress to the $G_1$/S boundary but prevent entry into S-phase. On release of this second block with deoxycytidine, all of the cells should progress through the cell cycle in a synchronized manner. To confirm that synchronization had occurred, synchronized cells were labelled with BrdU, sampled at 3-h intervals and processed using flow cytometry. This allowed quantification of the proportion of cells in each phase of the cell cycle at different times after release of the block. The effect of inclusion of 1 mM ALA (Sigma) in the culture media of synchronized cells during cell cycle progression was also studied.

The resultant synchronized DNA histograms were analysed using a curve-fitting program (ModFit LT, Verity Software House), which determined the percentage of cells in each phase of the cell cycle at each time point. (Five repeats of each experiment were performed.)

### PpIX determination

This method has been described in a previous publication (Wyld et al, 1997). In brief, flasks of synchronized cells were obtained as above. At times corresponding to S-phase, $G_2$/M and $G_1$, cells were incubated with 1 mM ALA in complete medium for 1 or 4 h, in the dark, at 37°C. PpIX concentrations in the cells and the media were then determined by spectrofluorimetry (excitation at 406 nm, emission at 604 nm, Perkin-Elmer LS-3 fluorescence spectrometer). Values were expressed as either ng PpIX per $10^5$ cell or ng PpIX per μg cellular protein. The cell count was determined using haemocytometry (Freshney, 1987) and the protein content by the micro-Lowry method (Lowry et al, 1951). Six triplicate repeats were performed.

### MTT assay

This technique allows quantification of cell survival after a cytotoxic insult. MTT (3-(4,5-dimethylthiazol-2yl)-2,5-diphenyl tetrazolium bromide, Sigma Chemicals, UK) is metabolized by the mitochondrial enzyme, lactate dehydrogenase to a violet-coloured formazan product in direct proportion to the number of cells present over a predetermined range (Stratford and Stephens, 1989). The optical density of the product is quantified spectrophotometrically on a 96-well-plate reader. MTT, 0.5 mg $ml^{-1}$, was added to each well of a 96-well plate and incubated for 2 h. The medium was then removed and replaced with 200 μl of dimethyl sulphoxide (DMSO) (Sigma, UK) to solubilize the formazan. The plate was then read on an ELISA plate reader (Anthos Labtec Instruments) at 550 nm absorbance. The initial plating density was determined by plating serial cell dilutions into the wells of a 96-well plate and comparing the direct haemocytometer count with the colour density produced. All further experiments used a cell density within the linear portion of the curve.

© Cancer Research Campaign 1998

### PDT sensitivity

This method has been described in a previous publication (Wyld et al, 1997). Cells were plated into 96-well plates at a cell density of $2 \times 10^4$ $ml^{-1}$ and synchronized as above. At times corresponding to S-phase, $G_2$ and $G_1$ of the cell cycle, 1 mM ALA was added to the cells and incubated for 1 or 4 h in the dark at 37°C. The cells were then exposed to violet light (350–450 nm), at a total dose of 0.5 J $cm^{-2}$ or 1.5 J $cm^{-2}$. The medium was then replaced with standard complete medium, the cells were returned to the incubator for 24 h and then an MTT assay was performed. Controls for light alone, ALA alone and neither light nor ALA were also performed. Percentage survival was determined according to the following equation:

$$\text{Survival } (\%) = \frac{\text{Optical density of treated well}}{\text{Optical density of control well}} \times 100$$

Six triplicate repeats were performed.

### Dual-labelling of unfixed, unsynchronized cells

Unsynchronized, exponentially growing cells were incubated with 1 mM ALA for either 1 or 4 h plus 10–25 μg $ml^{-1}$ bisbenzamide (Hoechst 33342, Sigma Chemicals, UK) in complete media for 1 h. The cells take up bisbenzimide in proportion to their DNA content (Crissman, 1995). The cells were then trypsinized and resuspended in media at a density of $1 \times 10^6$ cells $ml^{-1}$ and analysed on the flow cytometer (Beckton Dickinson, FACS Vantage). PpIX excitation was at 488 nm and emission at 600 nm and bisbenzamide excitation at 300 nm (ultraviolet) and emission at 500–550 nm. This produced a bivariate histogram of PpIX fluorescence (on the $y$-axis) against DNA (HO33342) fluorescence (on the $x$-axis). The mean PpIX fluorescence at DNA contents corresponding to $G_1$, S-phase and $G_2$ were then calculated. Six repeats of this experiment were performed for both 1- and 4-h ALA incubations.

### Statistical analyses

All data are represented as means plus or minus the standard error of the mean (SEM). Statistical analyses were carried out using an initial analysis of variance (Kruskal–Wallis), followed by a Mann–Whitney $U$-test. Statistical significance was accepted if $P < 0.05$.

### RESULTS

### Cell characteristics

### Cell cycle progression

Using BrdU pulse labelling the duration of the different phases of the cell cycle were calculated to be: S-phase 6 h; $G_2$ 3 h and $G_1$ 6 h with an overall cell cycle duration of 15 h. This is in agreement with the population doubling time (15.1 ± 2.9 h) previously determined in this laboratory.

### Cell synchronization

The mean percentage of cells in each phase of the cell cycle at each time point as calculated by ModFit analysis of the DNA

© Cancer Research Campaign 1998

**Figure 4** Bivariate histogram of PpIX fluorescence (y-axis) against DNA content (bisbenzamide fluorescence) after a 4-h incubation with ALA

histograms are shown in Figure 1. From these data, the optimal sampling times for each phase of the cell cycle after release of the thymidine block were determined. The percentage of BrdU-labelled cells can also be used as an estimate of the efficiency of synchronization in the S-phase period of the cell cycle, i.e. the 1- and 3-h samples. The percentage of BrdU-labelled cells at 1 and 3 h were 71 ± 3.3 and 77 ± 2.8, respectively, suggesting a good level of cell synchronization. The total cell cycle duration was 15 h. The 4-h ALA treatment times were as follows: S-phase from 1 to 5 h, $G_2$ from 5 to 9 h and $G_1$ from 10 to 14 h. For 1-h ALA treatment, the S-phase was from 1 to 2 h, $G_2$ from 5 to 6 h and $G_1$ from 11 to 12 h. Incubation of cells in media containing 1 mM ALA for the 15-h duration of this experiment had no detectable effect on cell cycle progression (data not shown).

### Protoporphyrin IX generation

After incubation of synchronized cells with ALA for 1 h, S-phase cells produced significantly more intracellular PpIX per $10^5$ cells than $G_2$ or $G_1$ cells (15.5 vs 8.1, $P < 0.05$ and 6.6, $P < 0.05$ respectively) and significantly more total (cellular plus media PpIX) PpIX $10^5$ cells than $G_2$ and $G_1$ cells (17.8 v 11, $P < 0.05$ and 9.6, $P < 0.05$ respectively, Figure 2). When PpIX generation was calculated per μg of cellular protein, a similar pattern was seen with intracellular PpIX being significantly greater in S-phase cells than in $G_2$ or $G_1$ cells (0.12 vs 0.98, $P < 0.05$, and 0.1, $P < 0.05$ respectively).

After a 4-h incubation of synchronized cells with ALA, total PpIX per $10^5$ cells was significantly less in $G_1$ than in $G_2$ and S-phase cells (39.5 vs 56, $P < 0.05$ and 51, $P < 0.05$ respectively), and intracellular PpIX was significantly less in $G_1$ than in S-phase cells (9.5 vs 12.7, $P < 0.05$ respectively, Figure 2). When PpIX was calculated per μg of protein after a 4-h incubation with ALA, no significant differences were noted between cell cycle phases (data not shown).

### PDT sensitivity

After a 1-h incubation with ALA and light administration, cell survival was greatest in $G_1$ cells and least with cells in $G_2$ at both light doses studied (Figure 3). With the 0.5 J $cm^{-2}$ light dose, $G_1$ survival was significantly greater than $G_2$ and S-phase (76.2 vs 44.2, $P < 0.05$ and 49.7, $P < 0.05$ respectively). At the higher light dose no significant differences were observed. After a 4-h incubation with ALA followed by PDT, no significant difference was noted between cell cycle phases (data not shown). Controls for light alone and ALA alone showed no significant toxicity compared with the no light, no ALA control.

### Dual labelling

After a 1-h incubation with ALA and bisbenzimide, DNA histograms were obtained (mean coefficient of variation of the $G_1$ peak of 10.3 ± 0.9) and PpIX fluorescence was detected. The bivariate histograms showed the $G_1$ cells to have a wider range of PpIX fluorescence than the other two phases of the cell cycle with a significantly lower mean ($G_1$, 24.8; S, 32.7; $P < 0.05$ and $G_2$, 35.4, $P < 0.05$). After a 4-h incubation with ALA and 1 h with bisbenzimide, these cell cycle phase differences were similarly significantly different ($G_1$, 46.5; S-phase, 55.9, $P < 0.05$; and $G_2$, 65.1, $P < 0.05$). The PpIX levels were significantly ($P < 0.05$) greater after a 4 h ALA incubation than after 1 h (55.2 ± 4.7 and 28.1 ± 0.7 respectively). An example of a PpIX/bisbenzamide histogram is shown in Figure 4 and mean cell cycle-specific PpIX fluorescence values are shown in Figure 5.

© Cancer Research Campaign 1998 

© Cancer Research Campaign 1998

is a broad range of PpIX fluorescence values, from 10 to 50 (arbitrary units), whereas the other two cycle phases have ranges from 30 to 50. The low fluorescent subpopulation of $G_1$ may be a PDT-resistant subgroup responsible for the increased percentage survival of $G_1$ cells after PDT.

In summary, it is possible that in a homogeneous population of tumour cells those cells in $G_1$ may be relatively insensitive to ALA-induced PDT (due to both decreased PpIX production and decreased PDT sensitivity) and thus survive treatment to provide foci of recurrence. However, there are many other confounding factors in the in vivo tumour microenvironment that may render cellular PpIX generation less efficient than in vitro, including pharmacokinetic bioavailability of ALA, poor tumour vascularity and hypoxia, which slows the rate of PpIX generation (L Wyld, unpublished data; Falk et al, 1959). It is also known that some of the toxicity of PDT may be due to microcirculatory collapse in the treated tissue, which may damage cells by inducing hypoxia (Reed et al, 1989), even if they have generated insufficient PpIX to suffer direct PDT toxicity. If PDT sensitivity, due to cell cycle variation, is a factor contributing to tumour recurrence, it could be overcome by the application either of adequate drug and light dosimetry or of a multiple-treatment regime to ensure cell cycle redistribution and tumour reoxygenation. This would allow relatively resistant $G_1$ cells to progress through the cell cycle to more sensitive cell cycle phases before administration of a second dose. At present, no data are available on whether cell cycle phase variation in PpIX generation or PDT sensitivity exists in vivo. Further in vivo studies are needed to validate these in vitro findings.

### ACKNOWLEDGEMENTS

Calibration of the light delivery system was performed by M Davis, Department of Medical Physics, Royal Hallamshire Hospital, Sheffield, UK. This work was supported by the Trustees of the Former United Sheffield Hospitals and the Yorkshire Cancer Research Fund.

### REFERENCES

Berlin NI, Neuberger A and Scott JJ (1956) The metabolism of δ-aminolaevulinic acid. 1. Normal pathways studied with the aid of [15]N. *Biochemistry* **64**: 80–90

Brown NC, Eliasson R, Reichard P and Thelander L (1969) Spectrum and iron content of protein B2 from ribonucleoside diphosphate reductase. *Eur J Biochem* **9**: 512–518

Churchill JR and Studzinski GP (1970) Thymidine as synchronising agent. iii. Persistence of cell cycle patterns of phosphatase activities and elevation of nuclease activity during inhibition of DNA synthesis. *J Cell Physiol* **75**: 297–304

Crissman HA (1995) Cell cycle analysis by flow cytometry. In *Cell Growth and Apoptosis*, 2nd edn. Studzinski GP (ed), pp. 21–43. IRL Press: Oxford

Christensen T, Feren K, Moan J and Pettersen E (1981) Photodynamic effects of haematoporphyrin derivative on synchronised and asynchronous cells of different origin. *Br J Cancer* **44**: 717

D'Hallewin MA and Baert L (1994) Long term results of whole bladder wall photodynamic therapy for carcinoma *in situ* of the bladder. *Urology* **45**: 763–767

Dolbeare F and Selden JR (1994) Immunochemical quantitation of bromodeoxyuridine: application to cell cycle kinetics. *Methods Cell Biol* **41**: 297–316

Dougherty TJ, Kaufman JE, Goldfarb A, Weishaupt KR, Boyle D and Mittleman A (1978) Photoradiation therapy for the treatment of malignant tumours. *Cancer Res* **38**: 2628–2635

Falk JE, Porra RJ, Brown A, Moss F and Larminie HE (1959) Effect of oxygen tension on haem and porphyrin biosynthesis. *Nature* **184**: 1217–1219

Freshney RI (1987) *Culture of Animal cells. A Manual of Basic Techniques*, 2nd edn, pp. 227–229. Alan R Liss: New York

Fukuda H, Batlle AMC and Riley PA (1993) Kinetics of porphyrin accumulation in cultured epithelial cells exposed to ALA. *Int J Biochem* **25**: 1407–1410

Gaullier JM, Geze M, Santus R, Melo TSE, Maziere J-C, Bazin M, Morliere P and Dubertret L (1995) Subcellular localisation of and photosensitisation by protoporphyrin IX in human keratinocytes and fibroblasts cultivated with 5-aminolaevulinc acid. *Photochem Photobiol* **62**: 114–122

Gomer CJ (1980) DNA damage and repair in CHO cells following haematoporphyrin irraditaion. *Cancer Letters* **11**: 161–167

Hayata Y, Kato H, Okitsu H, Kawaguchi M and Konaka C (1985) Photodynamic therapy with haematoporphyrin derivative in cancer of the upper gastrointestinal tract. *Sem Surg Oncol* **1**: 1–11

Hewitt HB and Wilson CW (1959) The effect of tissue oxygen tension on the radiosensitivity of leukaemia cells irradiated in situ in the livers of leukaemic cells. *Br J Cancer* **13**: 675–684

Humphrey RM and Dewey WC (1965) Radiosensitivity of normal and 5-bromodeoxyuridine treated mammalian cells during different phases of the cell cycle. *Exp Cell Res* **39**: 483–495

Kaczmarek L (1986) Biology of disease: protooncogene expression during the cell cycle. *Laboratory Invest* **54**: 365–376

Kriegmair M, Baumgartner R, Lumper W, Waidelich R and Hofstetter A (1996) Early clinical experience with 5-aminolaevulinic acid for the photodynamic therapy of superficial bladder cancer. *Br J Urol* **77**: 667–671

Lowry OH, Rosebrough NJ, Farr AL and Randall RJ (1951) Protein measurement with the folin phenol reagent. *Biol Chem* **193**: 265–275

Ma LW, Moan J, Steen HB and Iani U (1995) Antitumour activity of photodynamic therapy in combination with mitomycin C in nude mice with human colon adenocarcinoma. *Br J Cancer* **71**: 950–956

Moan J and Sommer S (1985) Oxygen dependence of the photosensitising effect of haematoporphyrin derivative in NHIK 3025 cells. *Cancer Res* **45**: 1608–1610

Moan J, McGhie J and Jacobson P (1983) Photodynamic effects on cells *in vitro* exposed to haematoporphyrin derivative. *Photochem Photobiol* **37**: 599–604

Nocka KH and Pelus LM (1988) Cell cycle specific effects of desferoxamine on human and murine haematopoietic progenitor cells. *Cancer Res* **48**: 3571–3575

Neckers LM and Cossman J (1983) Transferrin receptor induction in mitogen stimulated human T lymphocytes is required for DNA synthesis and cell division and is regulated by interleukin 2. *Proc Natl Acad Sci USA* **80**: 3494–3498

Rasheed S, Gardner MB, Rongey RW, Nelson-Rees WA and Arnstein P (1977) Human bladder carcinoma: characterisation of two new tumour cell lines and search for tumour viruses. *J Natl Cancer Inst* **58**: 881–887

Reed MWR, Mullin AP, Anderson GL, Miller FN and Weiman TJ (1989) The effect of photodynamic therapy on tumour oxygenation. *Surgery* **106**: 94–99

Rittenhouse-Diakun K, Van Leengoed H, Morgan J, Hryhorenko E, Paszkiewicz G, Whitaker JE and Oseroff AR (1995) The role of transferrin receptor (CD71) in photodynamic therapy of activated and malignant lymphocytes using the haem precursor δ-aminolaevulinic acid. *Photochem Photobiol* **61**: 523–528

Schick E, Kaufman R, Ruck A, Hainzl A and Boehncke W-H (1995) Influence of activation and differentiation of cells on the effectiveness of photodynamic therapy. *Acta Derm Venerol* **75**: 276–279

Schneckenburger H, Ruck A, Bartos B and Steiner R (1988) Intracellular distribution of photosensitising porphyrins measured by video-enhanced fluorescence microscopy. *J Photochem Photobiol B: Biol* **2**: 355–363

Sibille A, Lambert R, Souquet J-C, Sabben G and Descos F (1995) Long term survival after photodynamic therapy for oesophageal cancer. *Gastroenterology* **108**: 337–344

Sinclaire WK and Morton RA (1966) X-ray sensitivity during the cell generation cycle of cultured chinese hamster cells. *Radiat Res* **29**: 450–474

Stein GS and Borun TW (1972) The synthesis of acidic chromosomal proteins during the cell cycle of HeLa S-3 cell. *J Cell Biol* **52**: 292–307

Stein GS, Stein JL, Lian TJ, Last TJ, Owen T and McCabe L (1995) Cell synchronisation as a basis for investigating control of proliferation in mamalian cells. In *Cell Growth and Apoptosis*, 2nd edn, Studzinski GP (ed), pp. 193–204 IRL Press: Oxford

Stratford IA and Stephens MA (1989) The differential hypoxic cytotoxicity of bioreductive agents determined *in vitro* by the MTT assay. *Int J Radiat Oncol Biol Phys* **16**: 973–976

Suzuki S, Nakamura S and Sakaguchi S (1987) Experimental study of intra-abdominal photodynamic therapy. *Lasers Med Sci* **2**: 195–203

Terasima T and Tolmach LJ (1963) Variations in several responses of HeLa cells to X-irradiation during the division cycle. *Biophys J* **3**: 11–33

Weishaupt KR, Gomer CJ and Dougherty TJ (1976) Identification of singlet oxygen as the cytotoxic agent in photo-inactivation of a murine tumour. *Cancer Res* **36**: 2326–2329

Wyld L, Burn JL, Reed MWR and Brown NJ (1997) Factors affecting aminolaevulinic acid-induced generation of PpIX. *Br J Cancer* **76**: 705–712